# Reliability of the Polish Pelvic Organ Prolapse/Urinary Incontinence Sexual Questionnaire (PISQ-12) and Assessment of Sexual Function before and after Pelvic Organ Prolapse Reconstructive Surgery—A Prospective Study

**DOI:** 10.3390/jcm10184167

**Published:** 2021-09-15

**Authors:** Aleksandra Kamińska, Katarzyna Skorupska, Agnieszka Kubik-Komar, Konrad Futyma, Joanna Filipczak, Tomasz Rechberger

**Affiliations:** 1 2nd Department of Gynecology, Medical University, 20-954 Lublin, Poland; kasiaperzylo@hotmail.com (K.S.); futymakonrad@mp.pl (K.F.); rechbert@yahoo.com (T.R.); 2 Department of Applied Mathematics and Computer Science, University of Life Science, 20-033 Lublin, Poland; agnieszka.kubik@up.lublin.pl; 3 Medical University of Lublin, 20-059 Lublin, Poland; joannafilipczak15@onet.pl

**Keywords:** PISQ-12, reliability, sexual function, pelvic organ prolapse

## Abstract

It is estimated that 31–44% of all patients with symptomatic POP and/or UI suffer from sexual dysfunction. We aimed to validate the PISQ-12 in pre-and postmenopausal women and to assess the sexual function before and after POP reconstructive surgery. One hundred and forty sexually active patients were hospitalized due to symptomatic POP and 50 healthy controls were enrolled into the study. The patients were asked to complete PISQ-12, the FSFI and Beck’s depression scale questionnaires twice. The Cronbach’s alpha (α) was used to estimate the internal consistency. The scores were compared using the Intraclass Correlation Coefficient (ICC). Improvement in the QoSL (quality of sexual life) was observed in each age group of women. Pre-menopausal patients’ QoSL was much better, both before and after surgery (29.62 and 34.64 points, respectively). The correlation between questionnaires before surgery was 0.63, and after was −0.76. The α value for the PISQ-12 was 0.83 before the procedure and 0.80 afterwards. In all the groups, the test–retest reliability was good—ICC = 0.72. Vaginal reconstructive surgeries improve the QoSL. The only demographic factor influencing the QoSL was the menopausal status. The Polish version of the PISQ-12 is a reliable and responsive instrument for assessing the sexual function in patients with diagnosed POP and/or UI.

## 1. Introduction

Pelvic organ prolapse (POP) is a common problem among women around the world. Due to the aging of the population, it is estimated that in the 40 upcoming years, the number of women with POP symptoms will increase to 46% [1].

The negative impact of POP and/or urinary incontinence (UI) on the sexual life of patients is manifested by a decreased interest in sex and less frequent intercourse, which results from disturbances in the feeling of desire, excitement, difficulties in having an orgasm, dyspareunia, and involuntary leakage of urine during intercourse in the case of accompanying symptoms of a UI. It is estimated that 31–44% of all patients with symptomatic POP and/or UI suffer from sexual dysfunction [2]. The authors suggest a close relationship between the vaginal prolapse, sexual functions and the perception of one’s body, concluding that inferior self-esteem and a sense of less sexual attraction negatively affect women’s interest in sex, regardless of the severity of the defect [3,4].

Decision on the treatment method of POP should be based on the patients’ expectations, their values and faith, emotions, anatomical and functional improvement as well as the impact on sexual functions after surgery. Based on changes in anatomy alone, it was found that there was no way to predict the quality of sexual life after assessing the length and width of the vagina. Some authors suggest that reconstructive procedures in the case of POP, despite healing the defect, contribute to colpostenosis, changes in the physiological axis of the vagina, dyspareunia and reduced lubrication caused, among others, by surgical damage to vessels and nerves and scar forming as well [5,6,7].

An assessment of the subjective perception of health, and the quality of life (QoL) associated with it, is necessary to correctly determine the needs and expectations of patients and to properly plan and choose treatment methods tailored to a specific person. In addition to a physical examination and assessment of anatomy, the functional aspects of POP should be assessed using validated questionnaires to create a complete plan of treatment. These tools have been translated into different languages; however, we must always take into account certain restrictions and regional and religious variability [8].

The popularity of questionnaires to establish the impact of POP disorders on women’s sexual health after validation has been increasing for about 20 years. The Pelvic Organ Prolapse/Urinary Incontinence Sexual Questionnaire (PISQ-12) is the only questionnaire created to assess the sexual function of patients with UI and/or POP dysfunction [9]. It is a shortened version of the PISQ-31 questionnaire presented in 2001 and subjected to validation into Polish in 2017 [10]. PISQ-12, in the population of women with POP disorders, was classified to grade A according to the International Continence Society (ICS) [11]. This questionnaire is used to assess sexual function in heterosexual patients with diagnosed POP and/or UI who have been sexually active over the last 6 months. It should not be used for patients who have no partner or are sexually inactive [9,10,12].

Bearing in mind that sexual function has a significant impact on the well-being of patients with POP both before and after treatment, we aimed to assess the reliability of the Polish version of PISQ-12 so it can be valuable for future use in daily medical practice. Moreover, since sex life is still taboo for many women, the short version is more accessible to patients, enabling honest answers to be obtained. We also assessed the sexual function in women before and after POP reconstructive surgery.

## 2. Materials and Methods

### 2.1. Translation

Three independent translators translated the original PISQ-12 into Polish. A native speaker then back translated it into English to ensure conceptual equivalence with the original PISQ-12 version. Ten sexually active women suffering from POP were tested using the Polish version of the questionnaire. No major issues were observed, and minor problems that arose were straightened out.

### 2.2. Sample Size

Subject-to-item ratio of at least 5:1 has been recommended during performing psychometrics and scale evaluation [13,14]. There were 12 items on this version on the PISQ-12. Therefore, a sample size of at least 60 was required to fulfill the above criteria and assess the correlation of the scales with the Female Sexual Function Index (FSFI) and objective measures of different PISQ-12s. Assuming an effect size of 0.30 and an alpha < 0.05, a sample size of 60 would enable the study to achieve a power > 90 [15,16].

### 2.3. Study Population and Study Design

The prospective study included one hundred and forty sexually active patients hospitalized in the Gynecology Department between October 2015 and February 2018 due to symptomatic POP and 50 healthy controls were invited to the study (Table 1). We considered sexually active women who had sexual intercourse 3–4 times per month. Polish was the native language of all patients within the study population. After signing the informed consent, socio-demographic data such as age, parity, body mass index (BMI) and menopausal status were taken. Patients from the study group were asked to complete PISQ-12, Female Sexual Function Index (FSFI) and Beck’s depression scale questionnaires twice—before and 6 months after the surgical treatment; patients from the control group completed questionnaires only once. Beck’s depression scale was completed to exclude patients who assessed the quality of sexual life as low, due to symptomatic depression. Finally, 121 patients from the study group and all women from the control group were included into final assessment. Patients were stratified according to their age: group 1—less than 40 years, group 2—41–50 years, group 3—51–60 years and group 4—more than 60 years. In this part of the study, only the answers of patients who fully completed the questionnaires were taken into account. The local bioethics committee approved the study.

### 2.4. Bias

The responses were collected through face-to-face interviews conducted by four experienced interviewers (two teams each comprising two interviewers). The questionnaire was written in Polish after translation. All questions in the questionnaire were clarified with all interviewers before the study date. The interviewers were instructed to ask questions exactly as stated in the questionnaire and provide only non-directive guidance. To minimize inter-observer variability in conducting the interview, all team members met after the questionnaire was piloted to agree on a common interpretation of the findings. 

### 2.5. Questionnaires

The PISQ-12 is a condition-specific questionnaire; hence, it has been used only to reliably evaluate sexually active women with POP and/or UI. PISQ-12 scores are calculated as total scores or can be reported as scores for individual questions. Of the 12 questions, 9 are general sexual-function questions and 3 directly pertain to women with UI/POP [9]. The subscale scores ranged from 0–4, with higher scores representing better sexual function; the negatively worded items must be reversed prior to scoring such that 0 = always and 4 = never. Hence, reverse scoring is used for items 5–12, such that higher scores are indicative of better sexual function. 

The FSFI is composed of 19 questions. These enable an assessment of sexual function over the previous 4 weeks. The subscale scores ranged from 0–6, with higher scores indicating better sexual function. Subjects obtaining a total PL-FSFI score of 27.50 or lower were considered to have sexual dysfunction [17].

Beck’s depression scale is applied in the diagnosis of depression. It is used for self-assessment of well-being. The scale consists of 21 questions, to which the patient answers herself. There are 4 variants of responses that are evaluated differently. Subsequent disparate responses correspond to the increased intensity of the symptoms; therefore, they are also, respectively, scored from 0 to 3 points [18].

### 2.6. Validity

Face/content validity was assessed by obtaining feedback from a group of 10 sexually active women suffering from POP, and from 3 doctors taking part in the study who were specialists in the Urogynecology field. They reviewed the PISQ-12 questionnaire and clarified the translation.

Criterion validity—The final Polish version of the PISQ-12 was subsequently administered to Polish-speaking sexually active women assessed with POP at a university-based urogynecology department in Poland. Polish versions of the FSFI were also administered in order to evaluate the criterion validity of the Polish PISQ-12. The results of FSFI application were compared using Spearman correlation coefficient to assess the criterion validity. 

The internal consistency of the PISQ-12 questionnaire was estimated by way of Cronbach’s alpha coefficient (α)—a value greater than 0.7 indicates high reliability.

Reliability was tested by applying the Intraclass Correlation (ICC), an index of repeatability, by utilizing statistical software R—an ICC ≥ 0.7 was considered acceptable [19].

### 2.7. Statisical Analysis

Statistical analysis was performed using STATISTICA version 13.1 software (StatSoft, Poland), as well as open source R software [20]. P values less than 0.05 were considered significant. Significance of differences of means between studied groups was assessed using the *t* test for independent samples (in the case of two levels of a grouping variable) and one-way ANOVA (at more levels of the grouping variable). Rejection of the null hypothesis in the second case resulted in multiple comparisons in the form of Tukey’s test.

## 3. Results

A detailed characterization of the patients included in the study is presented in Table 1.

The total score of the PISQ-12 questionnaire ranges from 0 to 48. The higher the score is, the better the quality of sexual life is. Table 2 shows the mean scores ± SD of the patients’ responses to the questionnaire. The PISQ-12 results show a statistically significant improvement in the QoL of patients after reconstructive surgery, compared with pre-operative self-assessment (t = 8.48, *p* < 0.001).

Based on the analysis of patients’ responses to the questions contained in the PISQ-12 questionnaire, an improvement in the quality of sex life in each age group was found. The highest initial scores were observed in the 41–50 age group (group two), while the highest results after surgery were obtained in the group of patients up to 40 years of age (group one). The detailed distribution of results in the age group 50–60 years (group three) and above 60 years (group four) is presented in Table 3. Based on statistical analysis, however, no significant differences were found between the study groups in the investigated population. The test also showed no significant differences in the results of the PISQ-12 questionnaire in different age groups before surgery (F = 1.80, *p* = 0.15). It is difficult to state unequivocally whether the patient’s age has a direct impact on the answers given when completing the PISQ-12 questionnaire, and thus on the assessment of the quality of sexual life after surgery, as the results obtained in the analysis are on the border of statistical significance (F = 2.69; *p* = 0.049568) and there is no significant difference between the examined groups based on the results of comparisons with the Tukey test.

Based on the statistical analysis, there were no significant differences in the assessment of the quality of sexual life after surgery depending on BMI, both before and after surgery (F = 0.34; *p* = 0.79 and F = 1.49; *p* = 0.22, respectively).

The conducted data analysis showed no statistically significant impact of the level of education on the assessment of the quality of sexual life before treatment and after surgery (F = 0.85; *p* = 0.47 and *p* = F = 0.56; *p* = 0.64, respectively).

Based on the analysis of the answers given in the PISQ-12 questionnaire, an increase in the number of points in each examined group was found, regardless of the route and number of deliveries (Table 4). In the studied population of women, depending on the route and number of deliveries, the test showed no statistically significant differences between the study groups. Using the one-way ANOVA test of variance, no significant difference was found in the results of the PISQ-12 questionnaire prior to surgery depending on the route and number of births completed (F = 0.11; *p* = 0.95). The highest number of points in the questionnaire was observed among women after a CC, the lowest after three or more VDs—27.50 and 26.73, respectively. There is also no statistically significant difference in the quality of sexual life after surgery depending on the route and number of deliveries (F = 0.32; *p* = 0.80).

Pre-menopausal patients’ sexual life was much better, as indicated by the PISQ-12 questionnaire results, both before and after surgery (29.62 and 34.64 points, respectively), but this group was almost half the size of the post-menopausal group. The statistical test showed that these means differed significantly (t = 2.34; *p* = 0.02 and t = 2.65, *p* = 0.009) (Table 5). This was the only demographic factor in which the differences statistically significantly affected the results of the PISQ-12 questionnaire, both before and after treatment.

The average value of points obtained in the FSFI questionnaire in patients in the control group was 27.98 ± 3.61, while in the patients with POP who qualified for surgical treatment, this figure was 20.09 ± 8.09. There was a statistically significant difference in the answers to the questions in the questionnaire, and thus, better results of the quality of sexual life in the patients in the control group compared to the patients who qualified for surgical treatment.

A statistically significant difference was shown in the responses to the FSFI questionnaire before and after surgery in favor of responses given by the patients after surgery (Z = 7.04; *p* < 0.001) (Table 2). 

Based on the results of the Wilcoxon pair order test, a statistically significant improvement in the quality of sexual life after surgery was found in all the domains of the FSFI questionnaire (Table 6).

When related to the PISQ-12 questionnaire results, higher scores in FSFI were achieved by the patients before menopause, both before and after surgery (22.27 and 26.18, respectively). As in the case of the PISQ-12 questionnaire, statistically significant differences in the quality of sexual life after surgery depends on menopausal status, H = 5.86, *p* = 0.01.

According to Beck’s depression scale, the higher the score is, the greater the patient’s mood decrease is. In order to verify the impact of the procedure on the well-being of the patients, a nonparametric Wilcoxon pair order test (Z = 4.39; *p* < 0.001) was used in the statistical analysis. The response range on the Beck depression scale before surgery was 0–35, mean 11.64, SD ± 6.46. After surgery, the range ranged from 0–24, mean 9.87, SD ± 5.34.

The convergent validity of the results from the PISQ-12 and FSFI questionnaires was assessed by determining the value of the Spearman correlation coefficient. The correlation between the questionnaires before qualification for surgery (T1) was 0.63, and after surgery (T2)—0.76, indicating good convergent validity.

The α value for the PISQ-12 questionnaire was 0.83 before the procedure and 0.80 afterwards. In all the groups, the test–retest reliability was good—ICC = 0.72.

## 4. Discussion

Sexuality is an important aspect of women’s lives regardless of age. In the age group over 65, roughly one-third of the respondents report having sex and an interest in sex [21]. When considering the issues of the quality of sex life, it is necessary to take into account not only genetic, social, hormonal, biological and emotional factors, but also relationships with a partner and sexual dysfunctions that may occur in men, which can often have a dominant effect on the degree of approval of their own sexuality. In addition, chronic diseases and some medications can affect sexual function [22,23,24].

Polish studies showed that 69.73% of all post-menopausal women declare problems with their sexual life [25]. According to other sources, 28% of all men and 39% of all women in the 40–80 age range mention at least one aspect of sex life that is the reason for the deterioration in the quality of sexual life [23]. 

The relationship between the symptoms of POP, sexual function and body image with the QoL of women is emphasized. It is worth emphasizing that 20% of all the patients with POP and/or UI declare that the reason for the lack of sexual activity is shame or discomfort, not only on their part, but also on the part of their partner [26].

After 57 years of age, 11.8–17.8% of all women declare pain during intercourse, no interest in sex—38.4–49.3%, problems with lubrication—35.9–43.6%, and lack of orgasm—32.8–38.2% [27]. Our study showed an improvement in the quality of sexual life after POP surgery, as measured by the responses to the FSFI questionnaire in the desire, stimulation, lubrication, orgasm, satisfaction and pain domains of the questionnaire.

Upon analyzing the demographic factors regarding the patients operated upon in our center, such as age, BMI, fertility, menopausal status and education, and their impact on the quality of sexual life, we found that the only factor significantly affecting the type of answers and the degree of satisfaction with sex life is menopausal status. The postmenopausal patients achieved statistically significantly lower results than the premenopausal patients did, both on the PISQ-12 and FSFI questionnaires.

Kim et al. came to similar conclusions as those presented in our study about the impact of menopause status on the quality of sex life. In a study of 143 women with POP, the authors, based on the scores obtained in the PISQ-12 and FSFI questionnaires, confirmed the significant impact of menopause on the sexual function of women with POP (*p* < 0.001) [28].

Weber et al. did not show a statistically significant difference in the quality of sex life between 80 patients with POP and/or SUI and 30 women without any pelvic floor dysfunction who served as a control group. It was found that the severity of POP based on the POP-Q scale affects the sexual activity of patients; however, the frequency of sexual intercourse or the level of satisfaction does not differ from the results of patients without POP/UI. Therefore, satisfaction from sexual life was affected by conflicts in relationships and aging, and the only predictive factor for the deterioration of sexual function was the age of the patients [29].

Although in our results the only factor with statistical significance was menopause, while no similar relationship was found in the age groups, we must take into account that both menopausal status and age of patients can be considered together. According to the literature, both older age and natural menopause have a negative effect on women’s sex life, especially in the aspect of decreased libido, feeling of desire, excitement, orgasm, as well as undertaking sexual activity in itself. Decreased levels of estrogen, in addition to the negative impact on the physical aspect of intercourse caused by a feeling of dryness and pain during intercourse, also affects openness and the desire to initiate intercourse [22].

In a study performed by Elenskaia et al., the effect of childbirth on POP and QoL was evaluated. The authors found that the stage of prolapse worsened 5 years after VD, but not after a CC. Furthermore, there was no impact of prolapse symptoms on QoL, despite the fact that women after VD were more likely to experience a worse general sex score in the Personal Assessment Questionnaire–Pelvic Floor (PAQ-PF) [30]. Our results showed the best quality of sexual life (the highest number of points in the PISQ-12 questionnaire) among women after a CC, the lowest after three or more VDs—27.50 and 26.73, respectively. Still, an increase in the number of points in the PISQ-12 questionnaire (clearly indicating an improvement in sexual life) in each examined group was found after POP surgery, regardless of the route and number of deliveries. As a cesarean section was only two cases in the study, we cannot conclude the superiority of cesarean section compared to vaginal delivery based on the numbers mentioned above.

We used Beck’s depression scale to determine the well-being and self-assessment of the patients with POP. Before surgery, the average result was 11.64 ± 6.46, while in healthy patients without POP, the result was 7.14 ± 5.63. This indicates a reduced self-esteem in the group of patients with POP and the negative impact of POP on the perception of own body image and well-being, which confirms the increased risk of depression. We also observed a statistically significant improvement in the well-being of patients after surgery compared to the state before treatment (*p* < 0.001). Of note, Freak-Poli et al. noticed that well-being, more than the identified lack of depression symptoms, affects the quality of sex life [31].

In this study, we compared the quality of sexual life in patients with POP before and after reconstructive surgery. The results obtained from the PISQ-12 and FSFI questionnaires demonstrated a statistically significant improvement in the QoL of patients after repair operations, compared with pre-operative self-assessment in the PISQ-12 questionnaire (the average pre- and post-treatment scores were 27.24 ± 7.85 and 32.43 ± 6.48, respectively; *p* < 0.001). Moreover, improvement was demonstrated in each domain of the FSFI questionnaire, i.e., in terms of desire and arousal, as well as lubrication, dyspareunia, and in the relationship with the partner.

Surprisingly, tHoen et al., in an analysis conducted on the basis of the answers given in the PISQ-12 questionnaire by 124 patients with pelvic floor disorders symptoms, did not show a statistically significant improvement in the quality of sex life after treatment (both conservative and surgical) compared to the period before treatment (*p* = 0.14) [12].

Tyagi et al., on assessing sexual function in a group of 200 women after surgery due to POP and/or UI based on the PISQ-12 questionnaire, found statistically significantly better results in the self-assessment of patients 6 months after surgery, without further improvement after 12 months [32].

In turn, Celik et al. also observed a statistically significant improvement in sexual function 6 months after surgery based on the PISQ-12 questionnaire in patients operated on for SUI and SUI with POP, as compared to the pre-operative assessment. However, they did not show any change for the better in the group of patients only with POP [33].

Furthermore, Glavind et al. showed a significant improvement in the quality of sex life six months after repair surgery using the vaginal native tissue repair technique. According to the study, 72% of all the patients had higher results than before surgery, 18% the same, and 10% lower [34].

Opposite to the results obtained by us, Rogers et al. did not observe any obvious improvement in the quality of sex life in a group of 269 patients 6 months after operations performed due to UI and/or POP. Accordingly, significantly statistically lower results obtained in PISQ-12 were obtained in the group of women who underwent reconstructive surgery due to POP. No higher scores were noted for the emotional–behavioral domain, but there was a clear improvement in the domain assessing physical functions [9]. With regard to this difference in outcome, one should be aware that the PISQ-12 questionnaire, as well as its longer version, only helps in the diagnosis of patients with POP coexisting sexual disorders, and therefore, may not be sufficient to detect sexual dysfunction after treatment [6].

The study results show that the Polish PISQ-12 version has good face validity—no major troubles arose during the application and most respondents found it ‘comprehensive’ and a ‘good’ measure.

The limitation of the current study is the lack of knowledge about potential chronic diseases and sexual dysfunction in the partners of the examined patients—which could affect the assessment of the quality of women’s sexual life. What is more, this is a single setting study on the Polish population for which the score in the FSFI questionnaire is lower than in other nationalities (the optimal PL-FSFI cutoff score was 27.50).

## 5. Conclusions

Repair procedures for defects in the vaginal walls and/or uterus improve the quality of female sex life. The only demographic factor influencing the quality of female sexual life was the female menopausal status. Repair operations, regardless of the type of surgery performed, have a positive effect on the well-being of patients, reducing the risk of depression symptoms. 

The Polish versions of the PISQ-12 are reliable and responsive instruments for assessing the sexual function in heterosexual patients with diagnosed POP and/or UI.

## Figures and Tables

**Table 1:** Demographic characteristics of the study group patients.

	Study Group	Control Group
Age Mean ± SD (years)	53.92 ± 8.74	51.26 ± 5.64
BMI Mean ± SD (kg/m^2^)	27.16 ± 3.69	26.08 ± 3.06
Menopausal status (before vs. after menopause)	39 vs. 82 (32% vs. 68%)	23 vs. 27 (46% vs. 54%)
Parity (number)	119-VD 2-CC	35-VD 15-CC
Education (number)	54—secondary 44—higher 16—vocationa l7—primary	21—secondary 18—higher 8—vocationa l3—primary
SUI (Number)	38	10

Herein, continuous variables are presented as the mean ± SD, categorical variables are presented as numbers and %. BMI—body mass index; VD—vaginal delivery; CC—cesarean section; SUI—stress urinary incontinence.

**Table 2:** Mean scores ±SD of the patients’ responses to the PISQ-12 questionnaire.

Questionnaire	N	MEAN	MEDIAN	MINIMUM	MAXIMUM	SD	*p*
PISQ-12 T1	121	27.24	27	9	43	7.85	<0.01
PISQ-12 T2	121	32.43	33	13	46	6.48	
FSFI (T1)	121	20.09	22.60	2	32.6	8.09	<0.01
FSFI (T2)	121	23.82	25.15	2	34.5	6.96	

PISQ-12—The Pelvic Organ Prolapse/Urinary Incontinence Sexual Questionnaire; SD—standard deviation; FSFI—Female Sexual Function Index; N—number of patients; T1—Time point before surgery; T2—Time point 6 months after surgery.

**Table 3:** The detailed distribution of the PISQ-12 results among patients stratified according to age.

	Age (Years)	N	Mean	Minimum	Maximum	SD
PISQ-12 (T1)	1—<40	9	26.56	9	42	8.50
PISQ-12 (T2)			35.11	30	41	3.52
PISQ-12 (T1)	2—41–50	26	28.77	15	40	7.87
PISQ-12 (T2)			32.27	22	42	6.01
PISQ-12 (T1)	3—51–60	55	28.13	11	43	8.15
PISQ-12 (T2)			33.49	13	46	6.94
PISQ-12 (T1)	4—>60	31	24.58	13	41	6.75
PISQ-12 (T2)			29.90	19	40	6.09

PISQ-12—The Pelvic Organ Prolapse/Urinary Incontinence Sexual Questionnaire; SD—standard deviation; T1—Time point before surgery; T2—Time point 6 months after surgery; N—number of patients.

**Table 4:** The values of the parameters for the distribution of results of the PISQ-12 questionnaire depending on the route and number of deliveries of patients.

	Route and Number of Labors	N	Mean	Minimum	Maximum	SD
PISQ-12 (T1)	CC	2	27.50	24	31	4.95
PISQ-12 (T2)			35.50	30	41	7.78
PISQ-12 (T1)	VD—1	13	27.46	15	38	7.81
PISQ-12 (T2)			33.54	21	42	6.51
PISQ-12 (T1)	VD—2	57	27.61	9	43	8.23
PISQ-12 (T2)			32.39	13	45	6.67
PISQ-12 (T1)	VD—>3	49	26.73	13	40	7.68
PISQ-12 (T2)			32.06	19	46	6.36

PISQ-12—The Pelvic Organ Prolapse/Urinary Incontinence Sexual Questionnaire; SD—standard deviation; T1—Time point before surgery; T2—Time point 6 months after surgery; N—number of patients; CC—caesarean section; VD—vaginal delivery.

**Table 5:** The values of the parameters of the PISQ-12 questionnaire distribution before and after surgery-depending on the menopausal status of patients.

	Menopausal Status	N	Mean	Minimum	Maximum	SD	*p*
PISQ-12 (T1)	Before	39	29.62	9	42	7.55	<0.01
PISQ-12 (T2)			34.64	22	45	5.20	
PISQ-12 (T1)	After	82	26.11	11	43	7.79	<0.01
PISQ-12 (T2)			31.38	13	46	6.79	

PISQ-12—The Pelvic Organ Prolapse/Urinary Incontinence Sexual Questionnaire; SD—standard deviation; T1—Time point before surgery; T2—Time point 6 months after surgery; N—number of patients.

**Table 6:** The values of the parameters of the domains of the Female Sexual Function Index (FSFI) before and after surgery—depending on the menopausal status of patients.

Domain	Z	*p*
Domain I (desire)	3.87	0.0001
Domain II (stimulation)	6.54	<0.001
Domain III (lubrication)	4.49	0.000007
Domain IV (orgasm)	5.71	<0.001
Domain V (satisfaction)	5.55	<0.001
Domain VI (pain)	4.57	0.000005

## Data Availability

The data presented in this study are openly available with the author.
